# Congenital heart disease in pregnancy and severe maternal morbidity: A distributed data network study

**DOI:** 10.1002/pmf2.70370

**Published:** 2026-08-04

**Authors:** Elizabeth B. Sherwin, Benjamin Martin, Xiao Xu, Anna Booman, Alyssa Howren, Kimberlee McKay, Sadaf Kazi, Sean O'Reilly, Katie S. Kolla, Khyzer Aziz, Katherine Bianco, Stephanie A. Leonard

**Affiliations:** ^1^ Division of Maternal‐Fetal Medicine and Obstetrics Department of Obstetrics and Gynecology Stanford University School of Medicine Stanford California USA; ^2^ Biomedical Informatics and Data Science The Johns Hopkins University School of Medicine Baltimore Maryland USA; ^3^ Department of Obstetrics and Gynecology Columbia University Irving Medical Center New York New York USA; ^4^ Department of Anesthesiology Perioperative, and Pain Medicine Stanford University School of Medicine Stanford California USA; ^5^ Department of Epidemiology and Population Health Stanford University School of Medicine Stanford California USA; ^6^ Department of Obsetrics and Gynecology Avera McKennan Hospital Sioux Falls South Dakota USA; ^7^ Department of Obstetrics and Gynecology Sanford School of Medicine University of South Dakota Sioux Falls South Dakota USA; ^8^ National Center for Human Factors in Healthcare MedStar Health Research Institute Columbia Maryland USA; ^9^ Department of Emergency Medicine Georgetown University Medical Center Washington District of Columbia USA; ^10^ Department of Cardiothoracic Surgery Stanford Children's Health Stanford California USA; ^11^ Department of Pediatrics The Johns Hopkins University School of Medicine Baltimore Maryland USA

**Keywords:** common data model, heart defects, congenital, data analytics, electronic health records, heart diseases, pregnancy, severe maternal morbidity

## Abstract

**Introduction:**

Pregnant people with congenital heart disease (CHD) are a growing patient population in obstetrics, yet evidence on the risk for severe maternal morbidity (SMM) has largely been limited to studies that lack specificity for CHD. We conducted this study to demonstrate the utility of distributed data networks for obstetric research and to characterize pregnant patients with CHD and their risk of SMM.

**Methods:**

The study included female patients aged 12–55 years who delivered between 2015 and 2026 at three US academic healthcare organizations. We used a distributed data network study design by developing analytical code, executing the code locally at each organization using its electronic health record (EHR) data in a common data model (CDM), then aggregating the results. This analytical approach is rigorous, is reproducible, and protects patient confidentiality by not sharing patient‐level data. The Observational Medical Outcomes Partnership (OMOP) CDM was used by each organization, and we used OMOP standard concepts to identify CHD and SMM. We categorized outcomes as SMM, non‐transfusion SMM, and cardiac SMM based on the CDC SMM index. We analyzed the incidence of the outcomes and estimated risk ratios (RRs) and Wald 95% confidence intervals (CIs) using contingency tables.

**Results:**

The prevalence of CHD in pregnancy was 2.20% among 166,033 deliveries across three healthcare organizations. A total of 19.30% of people with CHD had an acquired heart condition, compared to 2.14% of people without CHD. SMM occurred in 9.43% of deliveries to people with CHD compared to 4.53% among people without CHD (RR, 2.08; 95% CI, 1.88, 2.31), and non‐transfusion SMM occurred in 6.72% vs 2.55% of deliveries (RR, 2.63; 95% CI, 2.33, 2.98), respectively. Cardiac SMM represented 51.84% of non‐transfusion SMM among people with CHD compared to 16.18% among people without CHD.

**Conclusions:**

Our distributed data network study using EHR data from three geographically diverse US healthcare organizations found that one in 15 people with CHD had non‐transfusion SMM compared with one in 39 people without CHD. This study demonstrates the feasibility and utility of distributed data networks for obstetric research and provides novel evidence on maternal outcomes for pregnant patients with CHD.

## 1 INTRODUCTION

Pregnant people with congenital heart disease (CHD) are a growing patient population, as recent advances in treatment of CHD have led to increased survival to adulthood [1, 2, 3, 4]. Most population health research on heart disease and severe maternal morbidity (SMM) has combined CHD and acquired heart disease and therefore does not disentangle the risk conferred specifically among individuals living with CHD [5, 6]. The few studies that separate CHD from acquired conditions are limited to SMM at delivery hospitalizations and therefore do not capture all SMM events in the postpartum period [6, 7, 8].

Most population health studies of CHD use national or statewide administrative databases, as single healthcare organizations often face sample size limitations [6, 7, 8, 9]. Distributed data network studies may be particularly useful for research on rare obstetric conditions using routinely collected health data. In this recently developed study design, healthcare data are mapped onto a common data model (CDM). Standardized analytical code is written and run locally in different datasets, and then aggregated results are combined. This approach allows for systematic analysis across distinct real‐world health databases, without necessitating patient data to be shared outside of the organization. Many healthcare organizations have mapped their electronic health record (EHR) data into a CDM, the most common being the Observational Medical Outcomes Partnership (OMOP) CDM. The OMOP CDM was developed by the Observational Health Data Sciences and Informatics (OHDSI) program and has resulted in impactful research across multiple fields of medicine [10, 11].

The objectives of this study were to: (1) demonstrate the feasibility and utility of conducting a distributed data network study using the OMOP CDM in obstetrics and (2) characterize pregnant patients with CHD and assess their incidence of severe maternal morbidity (SMM).

## 2 MATERIALS AND METHODS

We conducted a systematic analysis across EHR databases mapped to the OMOP CDM from three academic healthcare organizations across the United States (one each from the northeast, mid‐Atlantic, and west coast). Analytical code was developed at the primary study institution and then executed locally at each organization. Data between 2015 and 2026 were included, with exact years of study inclusion varying by site depending on EHR initiation dates and data mapping status. Included patients were females aged 12–55 years with a vaginal or cesarean delivery procedure code (Appendix 1 in the Supporting Information) and continuous observation of at least 30 days before and 42 days after delivery.

The exposure for this study was maternal CHD prior to delivery. We defined CHD using OMOP standard concepts mapped from International Classification of Diseases, 10th Revision, Clinical Modification (ICD‐10‐CM) diagnosis codes [5]. We validated the standardized concepts by generating counts from source ICD‐10‐CM codes to identify missing concepts for inclusion and not applicable concepts for removal, as reviewed by subject matter experts. The final CHD concept set is shown in Appendix 2 in the Supporting Information.

The primary outcome was non‐transfusion SMM between 30 days prior to delivery through 42 days postpartum. SMM was defined based on the Centers for Disease Control and Prevention (CDC) list of indicators mapped onto OMOP standard concepts (Appendix 3 in the Supporting Information) [12]. Secondary outcomes included SMM with transfusion and cardiac SMM.

Demographic characteristics assessed included race, ethnicity, and age. Additionally, we used the same methods as described above to identify prepregnancy characteristics (body mass index ≥40 kg/m^2^, preexisting diabetes, asthma, chronic kidney disease, autoimmune or connective tissue disease, chronic hypertension, and acquired heart conditions) and pregnancy characteristics (multiple gestation, gestational diabetes, preeclampsia, preterm delivery, and cesarean delivery) (detailed in Appendix 4 in the Supporting Information).

Within each institution's EHR dataset, we calculated frequencies and percentages for demographic, prepregnancy, and pregnancy characteristics by CHD status. Among those with CHD, we calculated the frequency of each specific congenital cardiac diagnosis. We analyzed the incidence of SMM outcomes and calculated risk ratios (RRs) and Wald 95% confidence intervals (CIs) using contingency tables. We used a directed acyclic graph (Appendix 5 in the Supporting Information) to guide analytical decision‐making and noted that clinical characteristics, such as comorbid acquired heart disease, would be mediators between CHD and SMM, rather than confounders. Results from each site were then aggregated, and all analyses were performed in the ATLAS [13] platform for OMOP CDM data and Microsoft Excel. Additionally, we conducted a sensitivity analysis stratified by time period (2015–2019 vs. 2020–2026).

## 3 RESULTS

A total of 166,033 deliveries were included across three healthcare organizations: 55,949 at Site A, 72,106 at Site B, and 37,978 at Site C. Maternal CHD prevalence was 2.20% across all three sites (1.84% at Site A, 2.36% at Site B, and 2.41% at Site C). Prevalence of preexisting diabetes, asthma, chronic kidney disease, autoimmune or connective disorders, and chronic hypertension was higher among those with CHD compared to those without CHD (Table 1; Appendix 6 in the Supporting Information). A total of 19.30% of people with CHD had an acquired heart condition, compared to 2.14% of people without CHD. Incidence of multiple gestation, preeclampsia, preterm delivery, and cesarean birth was higher among people with CHD compared to those without CHD, while incidence of gestational diabetes was similar in both groups. Among people with CHD, the most common conditions were ventricular septal defect, atrial septal defect, and Tetralogy of Fallot (Table 2).

**TABLE 1:** Demographic, prepregnancy, and pregnancy characteristics of study population by congenital heart disease status.

	People with congenital heart disease	People without congenital heart disease
	*N* = 3648	*N* = 162,385
	*n* (%)	*n* (%)
**Race**		
Asian	358 (9.81)	27,534 (16.96)
Black or African American	435 (11.92)	20,501 (12.62)
White	1985 (54.41)	71,059 (43.76)
Other or unknown	870 (23.85)	43,291 (26.66)
Hispanic or Latina/e	766 (21.00)	34,519 (21.26)
**Age at delivery** ^a^		
20–24 y	280 (7.68)	9396 (5.79)
25–29 y	599 (16.42)	24,323 (14.98)
30–34 y	1187 (32.54)	34,138 (21.64)
35–39 y	1061 (29.08)	50,176 (30.90)
40–44 y	382 (10.47)	15,675 (9.65)
**Prepregnancy characteristics**		
BMI ≥ 40 kg/m^2^	213 (5.84)	8168 (5.03)
Preexisting diabetes	161 (4.41)	3976 (2.45)
Asthma	456 (12.50)	14,985 (9.23)
Chronic kidney disease	78 (2.14)	1575 (0.97)
Autoimmune or connective tissue disease	466 (12.77)	17,448 (10.74)
Chronic hypertension	464 (12.72)	12,774 (7.87)
Acquired heart condition	704 (19.30)	3479 (2.14)
**Pregnancy characteristics**		
Multiple gestation	152 (4.17)	4753 (2.93)
Gestational diabetes	419 (11.49)	17,493 (10.77)
Preeclampsia	350 (9.59)	12,414 (7.64)
Preterm delivery	228 (6.25)	6301 (3.88)
Cesarean delivery	1675 (45.92)	65,361 (40.25)

^a^ Age not reported for 12–19 and 45–55 due to small numbers.

**TABLE 2:** Prevalence of congenital heart conditions.

Congenital heart disease condition^a^	*N* = 3648	%
Ventricular septal defect	891	24.42
Atrial septal defect	549	15.05
Tetralogy of Fallot	418	11.46
Congenital insufficiency of aortic valve	271	7.43
Coarctation of aorta	268	7.35
Patent ductus arteriosus	245	6.71
Discordant ventriculoarterial connection	241	6.61
Hypoplastic left heart syndrome	186	5.10
Double outlet right ventricle	185	5.07
Patent foramen ovale	169	4.63
Congenital stenosis of pulmonary valve	155	4.25
Bicuspid aortic valve	144	3.95
Atrioventricular septal defect and common atrioventricular junction	122	3.34
Congenital stenosis of aortic valve	103	2.82
Congenital insufficiency of mitral valve	93	2.55
Congenital anomaly of aorta	87	2.38
Congenital anomaly of coronary artery	85	2.33
Congenital atresia of pulmonary valve	70	1.92
Persistent left superior vena cava	63	1.73
Congenital anomaly of pulmonary artery	59	1.62
Congenital anomaly of tricuspid valve	55	1.51
Ebstein's anomaly	52	1.43
Right aortic arch	50	1.37
Common arterial trunk (truncus arteriosus)	44	1.20
Discordant atrioventricular connection	36	0.99

^a^ People may be represented in multiple diagnosis groups. Diagnoses with small numbers are not shown in the table.

The primary outcome of non‐transfusion SMM occurred in 6.72% of deliveries to people with CHD compared to 2.55% among people without CHD (RR, 2.63; 95% CI, 2.33, 2.98). Secondary outcomes of SMM with transfusion occurred in 9.43% of deliveries with CHD versus 4.53% of deliveries without CHD (RR, 2.08; 95% CI 1.88, 2.31), and cardiac SMM occurred in 3.48% of deliveries with CHD versus 0.41% of deliveries without CHD (RR, 8.44; 95% CI 7.00, 10.17). Cardiac SMM represented 51.84% of non‐transfusion SMM cases among people with CHD compared to 16.18% of cases among people without CHD (Table 3; Appendix 7 in the Supporting Information).

**TABLE 3:** Risk of severe maternal morbidity among those with congenital heart disease compared to those without.

	People without congenital heart disease	People with congenital heart disease	
	*N* = 162,385	*N* = 3648	Risk ratio
	*n* (%)	*n* (%)	(95% confidence interval)
Severe maternal morbidity (SMM)^a^	7362 (4.53)	344 (9.43)	2.08 (1.88, 2.31)
Non‐transfusion severe maternal morbidity^a^	4140 (2.55)	245 (6.72)	2.63 (2.33, 2.98)
Cardiac severe maternal morbidity^b^	670 (0.41)	127 (3.48)	8.44 (7.00, 10.17)

Abbreviation: CDC, Centers for Disease Control and Prevention.

^a^ Following CDC, SMM was defined with and without blood product transfusion as an indicator.

^b^ Cardiac SMM included acute myocardial infarction, aneurysm, cardiac arrest, ventricular fibrillation, conversion of cardiac rhythm, and acute heart failure.

In the sensitivity analysis, the incidence of SMM with transfusion and non‐transfusion SMM was similar in both time periods among those with and without CHD (Appendix 8 in the Supporting Information). Incidence of cardiac SMM was similar in both time periods in the CHD group; however, among those without CHD, incidence of cardiac SMM increased from 0.30% in 2015–2019 to 0.50% in 2020–2026.

## 4 DISCUSSION

This study successfully demonstrated the feasibility of utilizing a distributed data network for obstetric research and generated evidence on the incidence of SMM among pregnant patients with CHD. Across three diverse US healthcare institutions, we found that patients with CHD had almost three times the risk of non‐transfusion SMM and two times the risk of SMM including transfusion within 30 days before delivery to 42 days postpartum, compared to patients without CHD. Cardiac SMM represented half of non‐transfusion SMM among those with CHD. These results highlight the increased risk among patients with CHD for SMM related to the cardiovascular system as well as other organ systems.

This study achieved a large sample size and geographic variation by combining aggregate results from three EHR datasets mapped to the OMOP CDM. For studies of pregnant patients with relatively rare exposures and outcomes, traditional single‐site studies often face challenges of sample size and generalizability, and multisite studies encounter risks and obstacles related to patient privacy and data sharing. Distributed data network studies overcome these challenges by facilitating reproducible analysis on standardized data locally at each site, with only aggregate data shared outside of the home institution.

Our findings support clinical recommendations that pregnant patients with cardiac disease be managed by a multidisciplinary team, and for patients with CHD, adult congenital cardiologists should be included when possible [14]. Additionally, cardiovascular risk screening in pregnancy or preconception is recommended, especially given the range of severity of CHD presentations [3, 14]. Notably, we found that 19% of individuals with CHD also had an acquired heart condition. Thompson et al. found that people with both CHD and a pulmonary circulatory condition had the highest risk of adverse medical outcomes compared to those with CHD alone [9]. This population of pregnant people with CHD and an acquired cardiac or pulmonary condition may warrant special attention, and future studies should investigate the compound risk among those with both congenital and acquired conditions.

Our work is strengthened by the inclusion of SMM events 30 days before delivery through 42 days postpartum, which includes antepartum and postpartum encounters beyond the delivery hospitalization. Boghossian et al. [15] found that that 31% of SMM and 49% of non‐transfusion SMM cases were added after including antepartum and postpartum hospitalizations in addition to the delivery hospitalization. Furthermore, Denoble et al. [6] found that those with lower risk cardiac disease who did not experience SMM during delivery hospitalization were at increased risk of SMM or death during a postpartum readmission. These findings highlight the importance of assessing SMM beyond the delivery hospitalization and the need for close postpartum follow‐up of patients with risk factors for SMM, such as CHD.

Limitations of this study include that the data were not collected for research purposes and, therefore, the validity of phenotype algorithms for exposures and outcomes is limited. While the geographic variation of our data increases the generalizability of our findings, all included organizations were academic centers and our findings may not be generalizable to non‐academic healthcare institutions. We were unable to stratify by type of CHD or history of lesion repair, and suggest that future studies examine SMM by CHD severity, such as by using the World Health Organization classification [3, 6, 16].

## 5 CONCLUSION

Our distributed network study of EHR data from three geographically diverse US healthcare institutions found that one in 15 people with CHD had non‐transfusion SMM compared with one in 39 people without CHD. This study demonstrates the feasibility of distributed data networks for obstetric research and provides novel evidence for counseling pregnant patients with CHD.

## CONFLICT OF INTEREST STATEMENT

Xiao Xu has received honoraria and travel funding from the AAGL for serving on a practice guideline committee. All other authors declare no conflicts of interest.

## ETHICS STATEMENT

Because data used from Stanford University and Columbia University were de‐identified and aggregate, their use was considered not human subjects research. This study was reviewed and approved by the Johns Hopkins Institutional Review Board (Protocol #IRB000453833).

## Supporting information



Supporting Information

## Data Availability

The data that support the findings of this study are not openly available due to reasons of patient confidentiality per data use regulations from the institutions providing the data for analysis.
