# Supplementary material for: Congenital heart disease in pregnancy and severe maternal morbidity: A distributed data network study
Source: Pregnancy (Hoboken). 2026 Aug 4;2(5):e70370. doi: 10.1002/pmf2.70370 (PMC13449514; doi:10.1002/pmf2.70370)
Supplement: Supplementary file 1 [file PMF2-2-e70370-s001.docx]

**Appendix 1. Delivery procedures concept set.**

| **Concept ID** | **Concept Name** | **Domain** | **Exclude** | **Descendants** |
| --- | --- | --- | --- | --- |
| 4114637 | Forceps delivery | Procedure | FALSE | TRUE |
| 2110315 | Routine obstetric care including antepartum care, cesarean delivery, and postpartum care | Procedure | FALSE | TRUE |
| 2110322 | Routine obstetric care including antepartum care, cesarean delivery, and postpartum care, following attempted vaginal delivery after previous cesarean delivery | Procedure | FALSE | TRUE |
| 2110307 | Routine obstetric care including antepartum care, vaginal delivery (with or without episiotomy, and/or forceps) and postpartum care | Procedure | FALSE | TRUE |
| 2110319 | Routine obstetric care including antepartum care, vaginal delivery (with or without episiotomy, and/or forceps) and postpartum care, after previous cesarean delivery | Procedure | FALSE | TRUE |
| 2110320 | Vaginal delivery only, after previous cesarean delivery (with or without episiotomy and/or forceps) | Procedure | FALSE | TRUE |
| 4015701 | Cesarean section | Procedure | FALSE | TRUE |
| 4165077 | Cesarean hysterectomy | Procedure | FALSE | TRUE |

**Appendix 2. Congenital heart disease concept set.**

| **Concept ID** | **Concept Name** | **Domain** | **Exclude** | **Descendants** |
| --- | --- | --- | --- | --- |
| 4245646 | Congenital septal defect of heart | Condition | FALSE | TRUE |
| 4246484 | Congenital hypoplasia of heart | Condition | FALSE | TRUE |
| 42538528 | Congenital anomaly of great vessel | Condition | FALSE | TRUE |
| 441950 | Common arterial trunk (truncus arteriosus) | Condition | FALSE | TRUE |
| 35615056 | Congenital anomaly of cardiac chamber | Condition | FALSE | TRUE |
| 4182328 | Congenital abnormality of arterial duct | Condition | FALSE | TRUE |
| 4322316 | Congenital anomaly of heart valve | Condition | FALSE | TRUE |
| 315069 | Congenital heart block | Condition | FALSE | TRUE |
| 4100815 | Dextrocardia | Condition | FALSE | TRUE |
| 434462 | Ventricular septal defect | Condition | FALSE | TRUE |
| 4289309 | Atrial septal defect | Condition | FALSE | TRUE |
| 432432 | Total anomalous pulmonary venous return | Condition | FALSE | TRUE |
| 257660 | Partial anomalous pulmonary venous connection | Condition | FALSE | TRUE |
| 4061819 | Aortopulmonary window | Condition | FALSE | TRUE |
| 4147787 | Supravalvar aortic stenosis | Condition | FALSE | TRUE |
| 321109 | Congenital anomaly of coronary artery | Condition | FALSE | TRUE |
| 4100733 | Discordant atrioventricular connection | Condition | FALSE | TRUE |
| 4033322 | Traumatic interventricular septal defect | Condition | TRUE | TRUE |
| 4119953 | Post-infarction ventricular septal defect | Condition | TRUE | TRUE |
| 36716866 | Acquired atrial septal defect | Condition | TRUE | TRUE |
| 43020582 | Acquired excessive restriction of interatrial communication | Condition | TRUE | TRUE |
| 3656142 | Acquired stenosis of supravalvar area | Condition | TRUE | TRUE |
| 42536633 | Acquired ventricular septal defect | Condition | TRUE | TRUE |
| 37398927 | Naxos disease | Condition | TRUE | TRUE |

**Appendix 3. Severe Maternal Morbidity concept set.**

| **Concept ID** | **Concept Name** | **Domain** | **Exclude** | **Descendants** |
| --- | --- | --- | --- | --- |
| 312327 | Acute myocardial infarction^1^ | Condition | FALSE | TRUE |
| 4332246 | Aneurysm^1^ | Condition | FALSE | TRUE |
| 197320 | Acute renal failure syndrome | Condition | FALSE | TRUE |
| 42600379 | ARDs - Acute respiratory distress syndrome | Condition | FALSE | TRUE |
| 4195694 | Acute respiratory distress syndrome | Condition | FALSE | TRUE |
| 435616 | Amniotic fluid embolism | Condition | FALSE | TRUE |
| 321042 | Cardiac arrest^1^ | Condition | FALSE | TRUE |
| 437894 | Ventricular fibrillation^1^ | Condition | FALSE | TRUE |
| 443700 | Eclampsia | Condition | FALSE | TRUE |
| 4078925 | Pulmonary edema | Condition | FALSE | TRUE |
| 442310 | Acute heart failure^1^ | Condition | FALSE | TRUE |
| 198307 | Shock due to anesthesia | Condition | FALSE | TRUE |
| 4020879 | Malignant hyperpyrexia due to anesthetic | Condition | FALSE | TRUE |
| 4112809 | Aspiration pneumonitis due to anesthesia during labor and delivery | Condition | FALSE | TRUE |
| 132797 | Sepsis | Condition | FALSE | TRUE |
| 196236 | Septic shock | Condition | FALSE | TRUE |
| 201965 | Shock | Condition | FALSE | TRUE |
| 26942 | Hemoglobin SS disease with crisis | Condition | FALSE | TRUE |
| 440417 | Pulmonary embolism | Condition | FALSE | TRUE |
| 4127886 | Hysterectomy | Procedure | FALSE | TRUE |
| 4195473 | Temporary tracheostomy | Procedure | FALSE | TRUE |
| 2805869 | Extracorporeal or Systemic Assistance and Performance @ Physiological Systems (Procedure)^1^ | Procedure | FALSE | TRUE |
| 2788036 | Respiratory Ventilation, Less than 24 Consecutive Hours | Procedure | FALSE | TRUE |
| 4051932 | Procedure for arrhythmia^1^ | Procedure | FALSE | TRUE |
| 436093 | Disseminated intravascular coagulation | Condition | FALSE | FALSE |
| 4233224 | Cardiac insufficiency during AND/OR resulting from a procedure^1^ | Condition | FALSE | TRUE |
| 441874 | Cerebral thrombosis | Condition | FALSE | FALSE |
| 443454 | Cerebral infarction | Condition | FALSE | FALSE |
| 372924 | Cerebral artery occlusion | Condition | FALSE | FALSE |
| 375557 | Cerebral embolism | Condition | FALSE | FALSE |
| 432923 | Subarachnoid hemorrhage | Condition | FALSE | FALSE |
| 376713 | Cerebral hemorrhage | Condition | FALSE | FALSE |
| 4024656 | Transfusion of blood product^2^ | Procedure | FALSE | TRUE |
| 2788714 | Transfusion of Autologous Plasma Cryoprecipitate into Peripheral Vein, Percutaneous Approach^2^ | Procedure | FALSE | FALSE |
| 2788715 | Transfusion of Nonautologous Plasma Cryoprecipitate into Peripheral Vein, Percutaneous Approach^2^ | Procedure | FALSE | FALSE |
| 2788712 | Transfusion of Autologous Fresh Plasma into Peripheral Vein, Percutaneous Approach^2^ | Procedure | FALSE | FALSE |
| 2788713 | Transfusion of Nonautologous Fresh Plasma into Peripheral Vein, Percutaneous Approach^2^ | Procedure | FALSE | FALSE |
| 2788718 | Transfusion of Autologous Frozen Red Cells into Peripheral Vein, Percutaneous Approach^2^ | Procedure | FALSE | FALSE |
| 2788719 | Transfusion of Nonautologous Frozen Red Cells into Peripheral Vein, Percutaneous Approach^2^ | Procedure | FALSE | FALSE |
| 2788716 | Transfusion of Autologous Red Blood Cells into Peripheral Vein, Percutaneous Approach^2^ | Procedure | FALSE | FALSE |
| 2788717 | Transfusion of Nonautologous Red Blood Cells into Peripheral Vein, Percutaneous Approach^2^ | Procedure | FALSE | FALSE |
| 2788706 | Transfusion of Autologous Whole Blood into Peripheral Vein, Percutaneous Approach^2^ | Procedure | FALSE | FALSE |
| 2788707 | Transfusion of Nonautologous Whole Blood into Peripheral Vein, Percutaneous Approach^2^ | Procedure | FALSE | FALSE |
| 2788710 | Transfusion of Autologous Frozen Plasma into Peripheral Vein, Percutaneous Approach^2^ | Procedure | FALSE | FALSE |
| 2788711 | Transfusion of Nonautologous Frozen Plasma into Peripheral Vein, Percutaneous Approach^2^ | Procedure | FALSE | FALSE |
| 2788722 | Transfusion of Autologous Platelets into Peripheral Vein, Percutaneous Approach^2^ | Procedure | FALSE | FALSE |
| 2788723 | Transfusion of Nonautologous Platelets into Peripheral Vein, Percutaneous Approach^2^ | Procedure | FALSE | FALSE |
| 2788726 | Transfusion of Autologous Fibrinogen into Peripheral Vein, Percutaneous Approach^2^ | Procedure | FALSE | FALSE |
| 2788727 | Transfusion of Nonautologous Fibrinogen into Peripheral Vein, Percutaneous Approach^2^ | Procedure | FALSE | FALSE |
| 2788682 | Transfusion of Nonautologous Plasma Cryoprecipitate into Peripheral Vein, Open Approach^2^ | Procedure | FALSE | FALSE |
| 2788683 | Transfusion of Autologous Red Blood Cells into Peripheral Vein, Open Approach^2^ | Procedure | FALSE | FALSE |
| 2788680 | Transfusion of Nonautologous Fresh Plasma into Peripheral Vein, Open Approach^2^ | Procedure | FALSE | FALSE |
| 2788681 | Transfusion of Autologous Plasma Cryoprecipitate into Peripheral Vein, Open Approach^2^ | Procedure | FALSE | FALSE |
| 2788686 | Transfusion of Nonautologous Frozen Red Cells into Peripheral Vein, Open Approach^2^ | Procedure | FALSE | FALSE |
| 2788684 | Transfusion of Nonautologous Red Blood Cells into Peripheral Vein, Open Approach^2^ | Procedure | FALSE | FALSE |
| 2788685 | Transfusion of Autologous Frozen Red Cells into Peripheral Vein, Open Approach^2^ | Procedure | FALSE | FALSE |
| 2788674 | Transfusion of Nonautologous Whole Blood into Peripheral Vein, Open Approach^2^ | Procedure | FALSE | FALSE |
| 2788673 | Transfusion of Autologous Whole Blood into Peripheral Vein, Open Approach^2^ | Procedure | FALSE | FALSE |
| 2788678 | Transfusion of Nonautologous Frozen Plasma into Peripheral Vein, Open Approach^2^ | Procedure | FALSE | FALSE |
| 2788679 | Transfusion of Autologous Fresh Plasma into Peripheral Vein, Open Approach^2^ | Procedure | FALSE | FALSE |
| 2788677 | Transfusion of Autologous Frozen Plasma into Peripheral Vein, Open Approach^2^ | Procedure | FALSE | FALSE |
| 2788690 | Transfusion of Nonautologous Platelets into Peripheral Vein, Open Approach^2^ | Procedure | FALSE | FALSE |
| 2788689 | Transfusion of Autologous Platelets into Peripheral Vein, Open Approach^2^ | Procedure | FALSE | FALSE |
| 2788694 | Transfusion of Nonautologous Fibrinogen into Peripheral Vein, Open Approach^2^ | Procedure | FALSE | FALSE |
| 2788693 | Transfusion of Autologous Fibrinogen into Peripheral Vein, Open Approach^2^ | Procedure | FALSE | FALSE |
| 2788938 | Transfusion of Nonautologous Plasma Cryoprecipitate into Central Vein, Open Approach^2^ | Procedure | FALSE | FALSE |
| 2788939 | Transfusion of Autologous Red Blood Cells into Central Vein, Open Approach^2^ | Procedure | FALSE | FALSE |
| 2788936 | Transfusion of Nonautologous Fresh Plasma into Central Vein, Open Approach^2^ | Procedure | FALSE | FALSE |
| 2788937 | Transfusion of Autologous Plasma Cryoprecipitate into Central Vein, Open Approach2 | Procedure | FALSE | FALSE |
| 2788942 | Transfusion of Nonautologous Frozen Red Cells into Central Vein, Open Approach^2^ | Procedure | FALSE | FALSE |
| 2788940 | Transfusion of Nonautologous Red Blood Cells into Central Vein, Open Approach^2^ | Procedure | FALSE | FALSE |
| 2788941 | Transfusion of Autologous Frozen Red Cells into Central Vein, Open Approach^2^ | Procedure | FALSE | FALSE |
| 2788930 | Transfusion of Nonautologous Whole Blood into Central Vein, Open Approach^2^ | Procedure | FALSE | FALSE |
| 2788929 | Transfusion of Autologous Whole Blood into Central Vein, Open Approach^2^ | Procedure | FALSE | FALSE |
| 2788934 | Transfusion of Nonautologous Frozen Plasma into Central Vein, Open Approach^2^ | Procedure | FALSE | FALSE |
| 2788935 | Transfusion of Autologous Fresh Plasma into Central Vein, Open Approach^2^ | Procedure | FALSE | FALSE |
| 2788933 | Transfusion of Autologous Frozen Plasma into Central Vein, Open Approach^2^ | Procedure | FALSE | FALSE |
| 2788946 | Transfusion of Nonautologous Platelets into Central Vein, Open Approach^2^ | Procedure | FALSE | FALSE |
| 2788945 | Transfusion of Autologous Platelets into Central Vein, Open Approach^2^ | Procedure | FALSE | FALSE |
| 2788950 | Transfusion of Nonautologous Fibrinogen into Central Vein, Open Approach^2^ | Procedure | FALSE | FALSE |
| 2788949 | Transfusion of Autologous Fibrinogen into Central Vein, Open Approach^2^ | Procedure | FALSE | FALSE |
| 2788970 | Transfusion of Autologous Plasma Cryoprecipitate into Central Vein, Percutaneous Approach^2^ | Procedure | FALSE | FALSE |
| 2788971 | Transfusion of Nonautologous Plasma Cryoprecipitate into Central Vein, Percutaneous Approach^2^ | Procedure | FALSE | FALSE |
| 2788968 | Transfusion of Autologous Fresh Plasma into Central Vein, Percutaneous Approach^2^ | Procedure | FALSE | FALSE |
| 2788969 | Transfusion of Nonautologous Fresh Plasma into Central Vein, Percutaneous Approach^2^ | Procedure | FALSE | FALSE |
| 2788974 | Transfusion of Autologous Frozen Red Cells into Central Vein, Percutaneous Approach^2^ | Procedure | FALSE | FALSE |
| 2788975 | Transfusion of Nonautologous Frozen Red Cells into Central Vein, Percutaneous Approach^2^ | Procedure | FALSE | FALSE |
| 2788972 | Transfusion of Autologous Red Blood Cells into Central Vein, Percutaneous Approach^2^ | Procedure | FALSE | FALSE |
| 2788973 | Transfusion of Nonautologous Red Blood Cells into Central Vein, Percutaneous Approach^2^ | Procedure | FALSE | FALSE |
| 2788962 | Transfusion of Autologous Whole Blood into Central Vein, Percutaneous Approach^2^ | Procedure | FALSE | FALSE |
| 2788963 | Transfusion of Nonautologous Whole Blood into Central Vein, Percutaneous Approach^2^ | Procedure | FALSE | FALSE |
| 2788966 | Transfusion of Autologous Frozen Plasma into Central Vein, Percutaneous Approach^2^ | Procedure | FALSE | FALSE |
| 2788967 | Transfusion of Nonautologous Frozen Plasma into Central Vein, Percutaneous Approach^2^ | Procedure | FALSE | FALSE |
| 2788978 | Transfusion of Autologous Platelets into Central Vein, Percutaneous Approach^2^ | Procedure | FALSE | FALSE |
| 2788979 | Transfusion of Nonautologous Platelets into Central Vein, Percutaneous Approach^2^ | Procedure | FALSE | FALSE |
| 2788982 | Transfusion of Autologous Fibrinogen into Central Vein, Percutaneous Approach^2^ | Procedure | FALSE | FALSE |
| 2788983 | Transfusion of Nonautologous Fibrinogen into Central Vein, Percutaneous Approach^2^ | Procedure | FALSE | FALSE |

^1^Included in cardiac SMM

^2^Excluded for non-transfusion SMM

**Appendix 4. Concepts for conditions included in Table 1.**

| **Condition Name (timeframe)** | **Concept IDs** | **Concept Name** | **Domain** | **Exclude** | **Descendants** |
| --- | --- | --- | --- | --- | --- |
| **Class III obesity (280 days before delivery up to delivery)** | 434005 | Morbid obesity | Condition | FALSE | TRUE |
|  | 4256640 | Body mass index 40+ - severely obese | Observation | FALSE | FALSE |
|  | 37311728 | Obese class III | Observation | FALSE | FALSE |
| **Preexisting diabetes (1825 days before delivery up to delivery)** | 43531007 | Pre-existing diabetes mellitus in pregnancy | Condition | FALSE | TRUE |
| **Asthma (1825 days before delivery up to delivery)** | 317009 | Asthma | Condition | FALSE | TRUE |
| **Chronic kidney disease (1825 days before delivery up to delivery)** | 46271022 | Chronic kidney disease | Condition | FALSE | TRUE |
|  | 192359 | Renal failure syndrome | Condition | FALSE | TRUE |
| **Autoimmune or connective tissue disease (1825 days before delivery up to delivery)** | 4209487 | Thrombophilia due to antiphospholipid antibody | Condition | FALSE | TRUE |
|  | 134442 | Systemic sclerosis | Condition | FALSE | TRUE |
|  | 257628 | Systemic lupus erythematosus | Condition | FALSE | TRUE |
|  | 254443 | Sjögren's syndrome | Condition | FALSE | TRUE |
|  | 4141144 | Relapsing febrile nodular nonsuppurative panniculitis | Condition | FALSE | TRUE |
|  | 255348 | Polymyalgia rheumatica | Condition | FALSE | TRUE |
|  | 4079978 | Overlap syndrome | Condition | FALSE | TRUE |
|  | 4009035 | Multifocal fibrosclerosis | Condition | FALSE | TRUE |
|  | 76790 | Hypermobility syndrome | Condition | FALSE | TRUE |
|  | 4083100 | Fasciitis with eosinophilia syndrome | Condition | FALSE | TRUE |
|  | 253549 | Disorder of connective tissue | Condition | FALSE | TRUE |
|  | 201606 | Crohn's disease | Condition | FALSE | TRUE |
|  | 40479837 | Chronic ulcerative colitis | Condition | FALSE | TRUE |
|  | 436642 | Behcet's syndrome | Condition | FALSE |  |
|  | 45765493 | Autoimmune lymphoproliferative syndrome | Condition | FALSE | FALSE |
|  | 4326738 | Autoimmune hypoparathyroidism | Condition | FALSE | TRUE |
|  | 4098292 | Antiphospholipid syndrome | Condition | FALSE | TRUE |
| **Acquired heart condition (up to 30 days before delivery)** | 4222174 | Rheumatic disease of mitral valve | Condition | FALSE | TRUE |
|  | 315282 | Rheumatic disease of aortic valve | Condition | FALSE | TRUE |
|  | 4175807 | Rheumatic disease of tricuspid valve | Condition | FALSE | TRUE |
|  | 4108234 | Multiple valve disease | Condition | FALSE | TRUE |
|  | 319825 | Rheumatic heart disease | Condition | FALSE | TRUE |
|  | 4158911 | Non-rheumatic heart valve disorder | Condition | FALSE | TRUE |
|  | 319845 | Pulmonary valve disorder | Condition | FALSE | TRUE |
|  | 4232337 | Valvular endocarditis | Condition | FALSE | TRUE |
|  | 440736 | Endocarditis associated with another disorder | Condition | FALSE | TRUE |
|  | 321319 | Cardiomyopathy | Condition | FALSE | TRUE |
|  | 312383 | Postpartum cardiomyopathy | Condition | TRUE | TRUE |
|  | 4185932 | Ischemic heart disease | Condition | FALSE | TRUE |
|  | 316999 | Conduction disorder of the heart | Condition | FALSE | TRUE |
|  | 4201023 | Congenital conduction defect | Condition | TRUE | TRUE |
|  | 315069 | Congenital heart block | Condition | TRUE | TRUE |
|  | 313792 | Paroxysmal tachycardia | Condition | FALSE | TRUE |
|  | 4068155 | Atrial arrhythmia | Condition | FALSE | TRUE |
|  | 4307356 | Cor pulmonale | Condition | FALSE | TRUE |
|  | 444031 | Chronic heart failure | Condition | FALSE | TRUE |
|  | 314383 | Myocarditis | Condition | FALSE | TRUE |
|  | 4108950 | Acquired cardiac septal defect | Condition | FALSE | TRUE |
|  | 4237062 | Mural thrombus of heart | Condition | FALSE | TRUE |
|  | 314658 | Cardiomegaly | Condition | FALSE | TRUE |
|  | 43021734 | Fetal cardiomegaly | Condition | TRUE | TRUE |
|  | 43020781 | Fetal cardiomyopathy | Condition | TRUE | TRUE |
|  | 43020798 | Fetal myocarditis | Condition | TRUE | TRUE |
|  | 43020958 | Stenosis of fetal pulmonary valve | Condition | TRUE | TRUE |
|  | 4170062 | Congenital cardiomegaly | Condition | TRUE | TRUE |
|  | 320835 | Tetralogy of Fallot | Condition | TRUE | TRUE |
|  | 43020957 | Regurgitation of fetal pulmonary valve | Condition | TRUE | TRUE |
|  | 4245227 | Congenital abnormal shape of pulmonary valve | Condition | TRUE | TRUE |
|  | 4108723 | Congenital abnormality of arterial valves | Condition | TRUE | TRUE |
|  | 40490515 | Congenital abnormality of pulmonary valve cusp | Condition | TRUE | TRUE |
|  | 4289152 | Congenital absence of pulmonary valve | Condition | TRUE | TRUE |
|  | 320206 | Congenital atresia of pulmonary valve | Condition | TRUE | TRUE |
|  | 36713473 | Congenital cataract, hypertrophic cardiomyopathy, mitochondrial myopathy syndrome | Condition | TRUE | TRUE |
|  | 42599733 | Congenital dynamic stenosis of pulmonary valve | Condition | TRUE | TRUE |
|  | 4070914 | Congenital fusion of pulmonary valve segment | Condition | TRUE | TRUE |
|  | 4070795 | Congenital fusion of pulmonic cusps | Condition | TRUE | TRUE |
|  | 4244027 | Congenital hypertrophy of pulmonary valve | Condition | TRUE | TRUE |
|  | 4244184 | Congenital hypoplasia of pulmonary valve | Condition | TRUE | TRUE |
|  | 4035467 | Congenital insufficiency of pulmonary valve | Condition | TRUE | TRUE |
|  | 319921 | Congenital pulmonary valve abnormality | Condition | TRUE | TRUE |
|  | 313005 | Congenital stenosis of pulmonary valve | Condition | TRUE | TRUE |
|  | 42599734 | Congenital subvalvular stenosis of pulmonary valve | Condition | TRUE | TRUE |
|  | 42599735 | Congenital supravalvular stenosis of pulmonary valve | Condition | TRUE | TRUE |
|  | 42599736 | Congenital valvular stenosis of pulmonary valve | Condition | TRUE | TRUE |
|  | 4126122 | Pulmonic stenosis and congenital nephrosis | Condition | TRUE | TRUE |
|  | 37398927 | Naxos disease | Condition | TRUE | TRUE |
|  | 43020564 | Recurrent ventricular component of atrioventricular septal defect after prior cardiovascular surgical procedure | Condition | TRUE | TRUE |
|  | 42536642 | Ventricular septal defect following procedure | Condition | TRUE | TRUE |
| **Multiple gestation (7 days before to 1 day after delivery)** | 432969 | Multiple pregnancy | Condition | FALSE | TRUE |
| **Gestational diabetes (112 days before delivery up to delivery)** | 4024659 | Gestational diabetes mellitus | Condition | FALSE | TRUE |
|  | 45757789 | Postpartum gestational diabetes mellitus | Condition | TRUE | FALSE |
| **Chronic hypertension (anytime up to delivery)** | 320128 | Essential hypertension | Condition | FALSE | TRUE |
|  | 141084 | Pre-eclampsia or eclampsia with pre-existing hypertension | Condition | FALSE | TRUE |
|  | 4289142 | Eclampsia added to pre-existing hypertension | Condition | FALSE | TRUE |
|  | 4311246 | Pre-existing hypertension in obstetric context | Condition | FALSE | TRUE |
|  | 312648 | Benign essential hypertension | Condition | FALSE | TRUE |
| **Preeclampsia (140 days before delivery to 2 days after delivery)** | 439393 | Pre-eclampsia | Condition | FALSE | TRUE |
| **Preterm delivery (1 day before to 1 day after delivery)** | 36712702 | Preterm labor with preterm delivery | Condition | FALSE | TRUE |
|  | 4086393 | Premature delivery | Condition | FALSE | TRUE |
| **Cesarean delivery (1 day before to 1 day after delivery)** | 4015701 | Cesarean section | Procedure | FALSE | TRUE |

**Appendix 5. Directed Acyclic Graph**

**Appendix 6. Demographic, pre-pregnancy, and pregnancy characteristics of study population by study site and congenital heart disease status.**

|  | **People with congenital heart disease** | | | **People without congenital heart disease** | | |
| --- | --- | --- | --- | --- | --- | --- |
|  | **Site A**  N=1,032  n (%) | **Site B**  N=1,702  n (%) | **Site C**  N=914  n (%) | **Site A**  N=54,917  n (%) | **Site B**  N=70,404  n (%) | **Site C**  N=37,064  n (%) |
| **Race**  Asian  Black or African American  White  Other or Unknown | 259 (25.1)  29 (2.8)  485 (47.0)  259 (25.1) | 78 (4.6)  176 (10.3)  940 (55.3)  508 (29.8) | 21 (2.3)  230 (25.2)  560 (61.3)  103 (11.3) | 19,722 (35.9)  1,692 (3.1)  21,174 (38.6)  12,329 (22.5) | 5,704 (8.1)  8,094 (11.5)  32,196 (45.7)  24,410 (34.7) | 2,108 (5.7)  10,715 (28.9)  17,689 (47.7)  6,552 (17.7) |
| **Hispanic or Latino** | 292 (28.3) | 437 (25.7) | 37 (4.0) | 11,099 (20.2) | 21,599 (30.7) | 1,821 (4.9) |
| **Age at delivery**^a^  20-24 y  25-29 y  30-34 y  35-39 y  40-44 y | 71 (6.9)  169 (16.4)  348 (33.7)  308 (29.8)  106 (10.3) | 133 (7.8)  251 (14.7)  521 (30.6)  520 (30.6)  190 (11.2) | 76 (8.3)  179 (19.6)  318 (34.8)  233 (25.5)  86 (9.4) | 2,497 (4.5)  7,936 (14.5)  21,615 (39.4)  17,530 (31.9)  4,467 (8.1) | 3,504 (5.0)  9,193 (13.1)  22.497 (32.0)  23,183 (32.9)  8,697 (12.4) | 3,395 (9.2)  7,194 (19.4)  13,501 (36.4)  9,463 (25.5)  2,511 (6.8) |
| **Pre-Pregnancy Characteristics** | | | | | | |
| **BMI >= 40 kg/m^2^** | 41 (4.0) | 124 (7.3) | 48 (5.3) | 1,057 (1.9) | 4,584 (6.5) | 2,527 (6.8) |
| **Preexisting diabetes** | 51 (4.9) | 56 (3.3) | 54 (5.9) | 1,093 (2.0) | 1,364 (1.9) | 1,519 (4.1) |
| **Asthma** | 136 (13.2) | 182 (10.7) | 138 (15.1) | 3,550 (6.5) | 6,269 (8.9) | 5,166 (13.9) |
| **Chronic kidney disease** | 34 (3.3) | 29 (1.7) | 15 (1.6) | 371 (0.7) | 710 (1.0) | 494 (1.3) |
| **Autoimmune or connective tissue disease** | 143 (13.9) | 203 (11.9) | 120 (13.1) | 5,302 (9.7) | 7,923 (11.3) | 4,223 (11.4) |
| **Chronic hypertension** | 115 (11.1) | 216 (12.7) | 133 (14.6) | 3,033 (5.5) | 5,638 (8.0) | 4,103 (11.1) |
| **Acquired heart condition** | 265 (25.7) | 295 (17.3) | 144 (15.8) | 1,067 (1.9) | 1,884 (2.7) | 528 (1.4) |
| **Pregnancy Characteristics** | | | | | | |
| **Multiple gestation** | 34 (3.3) | 78 (4.6) | 40 (4.4) | 1,287 (2.3) | 2,437 (3.5) | 1029 (2.8) |
| **Gestational diabetes** | 170 (16.5) | 160 (9.4) | 89 (9.7) | 7,530 (13.7) | 5,830 (8.3) | 4,133 (11.2) |
| **Preeclampsia** | 120 (11.6) | 146 (8.6) | 84 (9.2) | 3,883 (7.1) | 5,036 (7.2) | 3,495 (9.4) |
| **Preterm delivery** | 81 (7.8) | 111 (6.5) | 36 (3.9) | 2,128 (3.9) | 2,980 (4.2) | 1,193 (3.2) |
| **Cesarean delivery** | 400 (38.8) | 844 (49.6) | 431 (47.2) | 18,013 (32.8) | 33,024 (46.9) | 14,324 (38.6) |

^a^Age not reported for 12-19 and 45-55 due to small numbers

**Appendix 7. Severe maternal morbidity by study site and congenital heart disease status.**

|  | **People with congenital heart disease** | | | **People without congenital heart disease** | | |
| --- | --- | --- | --- | --- | --- | --- |
|  | **Site A**  N= 1,032  n (%) | **Site B**  N=1,702  n (%) | **Site C**  N=914  n (%) | **Site A**  N=54,917  n (%) | **Site B**  N=70,404  n (%) | **Site C**  N=37,064  n (%) |
| **Severe maternal morbidity**^a^ | 111 (10.8) | 146 (8.6) | 87 (9.5) | 1,962 (3.6) | 3,299 (4.7) | 2,101 (5.7) |
| **Non-transfusion severe maternal morbidity**^a^ | 83 (8.0) | 104 (6.1) | 58 (6.3) | 1,059 (1.9) | 1,982 (2.8) | 1,099 (3.0) |
| **Cardiac severe maternal morbidity**^b^ | 49 (4.7) | 48 (2.8) | 30 (3.3) | 204 (0.4) | 277 (0.4) | 189 (0.5) |

^a^Following CDC, SMM defined with and without blood products transfusion as an indicator

^b^Cardiac SMM included acute myocardial infarction, aneurysm, cardiac arrest, ventricular fibrillation, conversion of cardiac rhythm, and acute heart failure

**Appendix 8. Severe maternal morbidity by congenital heart disease status, stratified by time period.**

|  | **2015-2019** | | | **2020-2026** | | |
| --- | --- | --- | --- | --- | --- | --- |
|  | **People without congenital heart disease**  **N = 67,581**  **n (%)** | **People with congenital heart disease**  **N = 1,263**  **n (%)** | **Risk Ratio**  **(95% Confidence Interval)** | **People without congenital heart disease**  **N = 94,862**  **n (%)** | **People with congenital heart disease**  **N = 2,387**  **n (%)** | **Risk Ratio**  **(95% Confidence Interval)** |
| **Severe maternal morbidity**^a^ | 2,425 (3.59) | 107 (8.47) | 2.36  (1.96, 2.84) | 4,998 (5.27) | 241 (10.10) | 1.92  (1.69, 2.17) |
| **Non-transfusion severe maternal morbidity**^a^ | 1,445 (2.14) | 78 (6.18) | 2.89  (2.32, 3.60) | 2,722 (2.87) | 170 (7.12) | 2.48  (2.14, 2.88) |
| **Cardiac severe maternal morbidity**^b^ | 204 (0.30) | 44 (3.48) | 11.54  (8.37, 15.91) | 472 (0.50) | 84 (3.52) | 7.07  (5.63, 8.89) |

^a^Following CDC, SMM defined with and without blood products transfusion as an indicator

^b^Cardiac SMM included acute myocardial infarction, aneurysm, cardiac arrest, ventricular fibrillation, conversion of cardiac rhythm, and acute heart failure
